# Dietary intake, food pattern, and abnormal blood glucose status of middle-aged adults: a cross-sectional community-based study in Myanmar

**DOI:** 10.3402/fnr.v60.28898

**Published:** 2016-05-04

**Authors:** Hlaing Hlaing Hlaing, Tippawan Liabsuetrakul

**Affiliations:** 1 Department of Preventive and Social Medicine, University of Medicine-Mandalay, Mandalay, Myanmar; 2 Epidemiology Unit, Faculty of Medicine, Prince of Songkla University, Hat Yai, Songkhla, Thailand

**Keywords:** abnormal blood glucose status, dietary intake, factor analysis, food pattern, suburban, urban

## Abstract

**Background:**

Lifestyle changes, particularly dietary intake, had resulted in increasing trends of type-2 diabetes mellitus worldwide. However, dietary intake is diverse across country contexts. This study aimed to compare the dietary intake, food patterns, and blood glucose among middle-aged adults living in urban and suburban areas in Mandalay city, Myanmar, and explore their relationships.

**Methods:**

A cross-sectional community-based study was conducted during June–November 2014. Adults aged 35–64 were randomly selected and requested to record all food they ate in a 4-day diary. Fasting and 2-hour postprandial blood glucose values were measured over two consecutive days. Dietary intakes were calculated in terms of energy, macronutrients, glycemic index, and glycemic load, and food patterns were identified by factor analysis. The relationships between food pattern, dietary intake, and blood glucose were assessed.

**Results:**

Of 440 participants, dietary intake between urban and suburban residents was significantly different. Six food patterns were identified. There was no difference in fasting and 2-hour postprandial blood glucose between urban and suburban residents, but a strong correlation between fasting blood glucose and 2-hour postprandial blood glucose was found (correlation coefficient=0.8). Identification of abnormal blood glucose status using original fasting and converted 2-hour postprandial values showed substantial agreement (prevalence-adjusted bias-adjusted Kappa=0.8). Relationships between food patterns and blood glucose or abnormal blood glucose status were not found.

**Conclusion:**

Food patterns were associated with dietary intake, not with abnormal blood glucose status. Two-hour postprandial blood glucose was highly correlated with fasting blood glucose and may be used for identifying abnormal blood glucose status.

Lifestyle including diet is changed dramatically in the twenty-first century and the prevalence of non-communicable diseases (NCD), especially diabetes mellitus (DM), is increasing worldwide (1). Importantly, DM became more prevalent in younger age groups, among whom undiagnosed and complicated DM can lead into economic and health burden especially on middle-aged adults (2, 3). Likewise, undiagnosed DM is a significant public health problem in the Southeast Asia Region (4).

Nowadays, there is growing interest in the analysis of overall diet or dietary/food patterns because of its usefulness in the prevention and treatment of chronic diseases, including DM (5). However, diet and food patterns are different across countries and can be expressed in various descriptions such as energy, carbohydrate, fat, protein, glycemic index (GI), and glycemic load (GL) appropriately (6–17). The population-based cross-sectional studies of DM highlighted the differences of DM prevalence between urban, suburban, or rural areas (18–22). Although the relationship between glucose tolerance abnormalities and dietary factors in terms of dietary intake and food patterns has been investigated, the conclusions are still contradictory (7–17). These findings may be explained by various methodologies, such as the different techniques used for measuring diet, food patterns, or blood glucose; study design; or study population.

The measurement of venous blood glucose, at fasting or after 75g glucose load, is the cornerstone in diagnosis of DM and glucose intolerance; however, it is not feasible and convenient for field surveys or in community settings. It will be helpful to check the ability of 2-hour postprandial blood glucose after a normal meal in the identification of abnormal blood glucose status in addition to fasting blood glucose (FBG). At present, owing to the accuracy and technological improvement of glucose meters, capillary blood glucose is widely accepted as an alternative measure of venous blood glucose by applying an appropriate conversion factor (23). The dietary or food patterns of Asian populations are similar, relying mainly on rice and rice products as a staple food. Nevertheless, because dietary or food patterns have changed as a consequence of globalization and urbanization (24), it will be beneficial to investigate the relationship between diet, food patterns, and abnormal blood glucose status for the future planning of DM prevention and control strategies. Therefore, this study aimed to explore and compare the relationships between dietary intake, food patterns, and blood glucose among middle-aged adults living in urban and suburban areas of Mandalay city, Myanmar.

## Material and methods

### Study design and setting

A cross-sectional community-based study was conducted in Mandalay city, Myanmar, during June–November, 2014. The study protocol was approved by the Ethical Committee of Faculty of Medicine, Prince of Songkla University, Thailand and Ethical Committee on Medical Research, Department of Medical Research (Upper Myanmar), Myanmar.

### Study sample and sampling

Adults aged 35–64 years who had resided in the study area for at least one year were included. Those who were not available within the study period or unwilling to participate, pregnant women, and the illiterate were excluded. The sample size was calculated based on a 10% difference of mean blood glucose and two proportions of high-risk dietary intake between urban and suburban population. At least 219 participants from urban and suburban areas were required, given the mean FBG of Myanmar people reported by World Health Organization (WHO) to be 90 mg/dl (25), and given an estimated standard deviation of 37 mg/dl and 20% and 10% of high-risk dietary intake in urban and suburban population, respectively, considering type I error of 5% and type II error of 20%. The relationships aimed to explore, thus the sample size was not calculated.

There are five townships in Mandalay city of which each township is composed of wards and blocks. In order to achieve the random sampling of middle-aged adults for this study, a multistage sampling technique was used in both urban and suburban areas. The sampling frame of this study included two townships, six wards, and 30 blocks. Each township is made up of urban and suburban wards with slightly different proportion. Among five urban townships of Mandalay city, two townships were selected by simple random sampling, taking into consideration urban-suburban proportions and representativeness. The selected Township 1 has more proportion of urban, while the selected Township 2 has more proportion of suburban. Therefore, two urban wards and one suburban ward were selected from Township 1, and one urban and two suburban wards were selected from Township 2. Each ward has the same approximate number of blocks. Thus, five blocks in each ward were randomly selected.

In urban areas, the research team obtained the lists of households according to house number and family members within each household. Households containing any middle-aged adults were randomly selected. If more than one eligible adult lived in a selected household, only one was randomly chosen. The response rate in urban areas was 89%. In suburban areas, lists of households according to house number and family members were not available. Thus, the first household was randomly selected. After selecting the household, the research team checked whether one entitled middle-aged adult was available or not. If there were more than one eligible adult in any house, only one was chosen by means of availability and consent. Houses were visited until the required sample size was reached. The response rate in suburban areas was 85%. The interviews and measurements were conducted by research team under the facilitation of community leaders.

### Data collection

A research team visited household and invited an eligible middle-aged adult to participate in the study. On the first day, after signing the informed consent, they were interviewed using a structured questionnaire and provided a 4-day food diary and a bowl for measuring food. All participants were explained how to use the standard reference of food measures such as cup, glass and bowl. Each participant recorded all food intakes in the given diary. On Day 2 and Day 3, the research team visited again for obtaining either capillary fasting or 2-hour postprandial blood glucose after a normal habitual morning meal at fingertip prick by ACCU – CHEK ^®^ Performa, which has good precision and render less technical error given its user-friendly nature (26). Dietary intake was continually recorded in diary for 4 days and the research team collected the diary on Day 5.

Main outcome measures in this study were dietary intake, food patterns and blood glucose (FBG and 2-hour postprandial blood glucose after a normal habitual morning meal).

### Socio-demographic characteristics

Socio-demographic characteristics of the participants included age, sex, ethnicity, marital status, educational levels, professional activity, income, and family size. Education levels were classified as less than secondary school, secondary school, and higher than secondary school. Professional activity was classified as dependent/retired, laborer/worker (unskilled workers, skilled manual workers), and government/business (government employees, non-government employees, small business owners, business people).

### Dietary intake and food groups

A total of 1,760 person-days dietary records in the 4-day food diary were firstly transcribed in terms of energy (kcal), carbohydrate (g), fat (g), protein (g), GI (per day), and GL (per day). For an estimation of the intake of traditional meals and snacks, the Myanmar Food Composition Table (national unpublished data, 1993) was used. The Food Composition database for the INMUCAL program (27) published by Institute of Nutrition, Mahidol University, Thailand, was applied for data related to fruits, vegetables, and meat products. GI and GL values were derived from the International table of GI and GL values: 2002 (6). Average daily GL for each participant was assessed by summing the products of carbohydrate content per gram for each food item by the amount of food per day, times its GI, and divided by the total daily carbohydrate intake (10). All foods mentioned in participants’ diaries were grouped by their similarities (7) and 24 food subgroups were achieved as shown in Table 1.

**Table 1:** Food or food groups mentioned in 4-day food diary

Group	Food
Rice	White rice; steamed or fried
Sticky rice	Glutinous rice
Rice product	Rice noodle, traditional food made from rice
Wheat	Wheat noodle, food made from wheat, bread
Poultry	Chicken or birds, all preparations
Meat	Beef or mutton, steak, ground meat, or mixed dishes
Pork	Steak, ground pork, mixed dishes
Processed meat	Sausage, dry mutton, dry beef
Fish and fish roe	Fish (including fish roe) and prawn
Visceral meat	Chicken liver, gizzard, beef liver, etc.
Egg	All preparations
Salted	Dry salted fish, fish sauce, fish paste
Legume	Beans, peas, peanut
Vegetable	Green leafy vegetables, other vegetable, Potato, taro, sweet potato, vegetable salad
Preserved vegetable	Pickled vegetable
Fruit	Orange, apple, grapefruit, lime, lemon, banana, mango, watermelon, guava, papaya, pineapple, durian, other fruits, and freshly squeezed fruit juice
Milk	Milk and milk products
Soft drink	Carbonated drinks
Tea	Hot tea
Coffee	Hot and cold coffee
Sweet	Any kind of sweet food, sugar, condensed milk, jaggery
Deep fried	All kinds of deep-fried food
Fermented food	Fermented fish, other fermented food
Snack	Food consumed as snack like potato chips, fish cracker, sunflower seed

### Blood glucose concentration and abnormal blood glucose status

To adjust the values of capillary blood glucose and plasma glucose, a conversion factor of 1.11 was applied to the resulted values (23). The WHO stated that capillary blood glucose and plasma glucose can be assumed to be identical in fasting state (28, 29). In our study, original and converted values were recorded for investigation of the blood glucose level. Abnormal blood glucose status was classified by WHO 2006 criteria using original FBG and converted 2-hour postprandial blood glucose after a normal habitual morning meal (2hPPBG): FBG more than 126 mg/dl or 2hPPBG more than 200 mg/dl was defined as DM.

### Data management and analysis

The data were checked, coded, and double-entered in the Epidata version 3.1 and analyzed with R version 3.1.2 (R Foundation for Statistical Computing, Vienna, Austria) using an epicalc package. Since the multistage sampling was applied in our study, sampling weights was taken into account by survey analysis using a survey package to adjust the standard error and *p*-value. Socio-demographic characteristics, dietary intakes, food patterns, and blood glucose of people between urban and suburban areas were analyzed by t-test or Mann-Whitney test, as appropriate for continuous data and chi-square test or Fisher's exact test for categorical data. The relationship of blood glucose between fasting and 2-hour postprandial state was analyzed using correlation coefficient. Agreement between identification of abnormal blood glucose status employing original FBG and converted 2-hour postprandial blood glucose was observed by prevalence-adjusted and bias-adjusted kappa coefficient (PABAK).

The food pattern derivation was performed by factor analysis (10–14, 30). Sampling adequacy and the inter-correlation of variables were checked by Bartlett's test of sphericity and Kaiser-Meyer-Olkin. The number of factors determined was based on the Kaiser criterion (eigenvalues >1). Factor scores were interpreted as a correlation coefficient. Food groups with absolute factor loadings >0.3 were designated as a significant contributor to the pattern derived. A factor score for each food pattern, based on correlation of his or her dietary data with the groupings, was obtained. One food pattern was chosen on the basis of the highest factor score. Dietary intakes and abnormal blood glucose status across six food patterns were calculated by the Analysis of Variance (ANOVA) or Kruskal-Wallis test. A significance level was considered as *p*-value less than 0.05.

## Results

Table 2 shows the socio-demographic characteristics of 440 middle-aged adults residing in urban and suburban areas, adjusted by the sample weights. Three-fourths of them were female and currently married. Approximate half of participants had education of less than secondary school and one-third were dependent or retired. No significant difference was found based on these characteristics.

**Table 2:** Socio-demographic characteristics of participants after applying sampling weights

		Residence	
		
Characteristics	Total (*n*=440), mean% (SE)	Urban (*n*=220), mean% (SE)	Suburban (*n*=220), mean% (SE)
Age (years)			
Mean (SE)	49.0 (0.52)	49.5 (0.62)	48.7 (0.34)
Sex			
Male	32.3 (0.05)	25.7 (0.04)	36.8 (0.07)
Female	67.7 (0.05)	74.3 (0.04)	63.2 (0.07)
Ethnicity			
Bamar	92.8 (0.02)	93.0 (0.01)	92.6 (0.04)
Others	7.2 (0.02)	7.0 (0.01)	7.4 (0.04)
Marital status			
Never married	15.7 (0.02)	18.7 (0.06)	13.7 (0.01)
Currently married	71.3 (0.02)	68.9 (0.07)	72.9 (0.01)
Separated and widowed	13.0 (0.01)	12.4 (0.01)	13.4 (0.01)
Level of education			
Less than secondary School	48.8 (0.02)	49.5 (0.03)	48.4 (0.02)
Secondary school	25.2 (0.03)	20.0 (0.02)	28.6 (0.02)
Higher than secondary School	26.0 (0.03)	30.5 (0.04)	23.0 (0.01)
Professional activity			
Dependent/retired	33.1 (0.05)	36.8 (0.02)	30.6 (0.07)
Laborer/worker	39.7 (0.08)	32.2 (0.03)	44.9 (0.07)
Government/business	27.2 (0.03)	31.0 (0.04)	24.5 (0.01)
Income (US Dollars)			
Mean (SE)	230.9 (17.02)	255.3 (14.6)	215.6 (11.0)
Family size			
<5	43.0 (0.02)	40.1 (0.04)	45.0 (0.02)
≥5	57.0 (0.02)	59.9 (0.04)	55.0 (0.02)

**Fig. 1:**
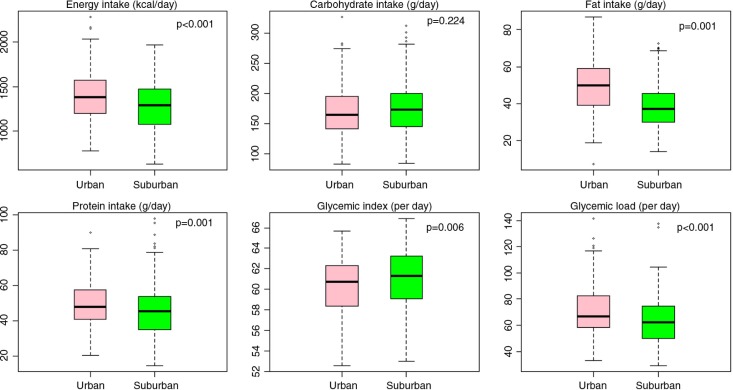
Average daily intake of energy, carbohydrate, fat, protein, glycemic index, and glycemic load among urban and suburban participants.

Figure 1 presents the average daily intake of energy, carbohydrate, fat, protein, GI, and GL among urban and suburban participants. All the dietary intakes, except carbohydrate, were significantly different between the urban and suburban participants. The means and standard deviations of daily total energy and fat in urban and suburban participants were 1,410 kcal (282.8) and 49.8 g (14.2), and 1,289.7 kcal (265.5) and 38.3 g (12.3), respectively. The medians and interquartile ranges of protein and GL of the participants in urban and suburban areas were 48 g (40.9, 57.5) and 66.9 per day (58.3, 82.1), and 45.5 g (35.2, 53.5) and 62.4 per day (50.2, 74.7), respectively. In contrast, the median of GI was 60.8 per day (58.4, 62.3) among those in urban area and 61.3 per day (59.1, 63.2) among those in suburban area.

Six food patterns identified from factor analysis, which explained 41% of total variance are shown in Table 3. The first factor was named as ‘Milk-sweets-wheat’ because it represented a high intake of milk and dairy products, sweets, and wheat products. The second factor, ‘Rice-vegetable-meat’ was characterized by rice, vegetable, pork, and chicken. The third factor was identified by legume, fermented food, preserved meat, and preserved vegetable and was given the name ‘Legume-preserved food’. The fourth factor loaded with snack, soft drink, and salted food was labeled ‘Snack-soft drink-salted’. The fifth factor, namely ‘Fruit-coffee-sticky’ denoted a high content of fruit, coffee, and sticky rice. The sixth one, characterized by high intake of deep-fried food and egg, was simply named ‘Deep-fried egg’.

**Table 3:** Factor loading matrix for major food patterns identified by factor analysis

Milk-sweets-wheat		Rice-vegetable-meat		Legume-preserved food		Snack-soft drink-salted		Fruit-coffee-sticky		Deep-fried egg	
					
Food	Loading	Food	Loading	Food	Loading	Food	Loading	Food	Loading	Food	Loading
Milk and milk products	0.863	Rice	0.572	Legume	0.501	Snack	0.472	Fruit	0.641	Deep-fried food	0.664
Sweets	0.862	Vegetable	0.542	Preserved meat	0.440	Soft drink	0.443	Coffee	0.626	Egg	0.397
Wheat product	0.311	Pork	0.348	Fermented food	0.441	Salted food	0.376	Sticky rice	0.496	Legume	0.307
		Chicken	0.329	Preserved vegetable	0.390	Visceral meat	0.316	Tea	−0.519	Rice product	−0.671
		Wheat product	−0.356	Egg	−0.417	Fish	−0.722				

Omitted from table were food groups with factor loading<±0.3 for all patterns (for simplicity).

Results of blood glucose concentrations and their classification were presented in Table 4. A strong relationship between fasting and 2-hour postprandial blood glucose was found. The identification of abnormal blood glucose status by using original FBG and converted 2-hour postprandial blood glucose showed substantial agreement after adjusting prevalence.

**Table 4:** Correlation between fasting and 2-hour postprandial blood glucose and identification of abnormal blood glucose status

	Correlation of fasting and 2-hour postprandial blood glucose concentration								
	
	Total			Urban			Suburban		
					
	Original, mean (sd)	Converted, mean (sd)	r	Original, median (IQR)	Converted, median (IQR)	r	Original, median (IQR)	Converted, median (IQR)	r
FBG	115.6 (47.37)	128.3 (52.58)	0.76	101 (93.8, 115)	112.1 (104.1, 127.7)	0.80	101 (94, 115.2)	112.1 (104.3, 127.9)	0.74
2hPP	145.9 (70.08)	161.9 (77.79)		124 (105, 147.2)	137.6 (116.6, 163.4)		124.5 (107, 155)	138.2 (118.8, 172.1)	

Agreement of abnormal blood glucose status by using original fasting and converted 2-hour postprandial blood glucose									

	Total			Urban			Suburban		
						
	DM	Non-DM	PABAK	DM	Non-DM	PABAK	DM	Non-DM	PABAK
	*n* (%)	*n* (%)		*n* (%)	*n* (%)		*n* (%)	*n* (%)	

DM	49 (68.1)	24 (6.5)	0.79	26 (72.2)	12 (6.5)	0.80	23 (63.9)	12 (6.5)	0.77
Non-DM	23 (31.9)	344 (93.5)		10 (27.8)	172 (93.5)		13 (36.1)	172 (93.5)	

Agreement of blood glucose status in lower part of the table, column values are classified by original fasting blood glucose and row values by converted 2-hour postprandial blood glucose. FBG=fasting blood glucose; 2hPP=2-hour postprandial blood glucose; Original=values of blood glucose concentration before applying conversion factor (original value); Converted=values of blood glucose concentration after applying conversion factor; r=correlation coefficient; PABAK=prevalence-adjusted and bias-adjusted kappa; DM=diabetes mellitus; Non-DM=non-diabetes mellitus; sd=standard deviation; IQR=interquartile range.

Dietary intakes and abnormal blood glucose status across different types of food patterns are presented in Table 5. The food patterns among urban and suburban participants were significantly different but, after adjusting the standard errors for sampling design, no significant association was found. Among urban respondents, the rice-vegetable-meat pattern and fruit-coffee-sticky patterns were most commonly found (22.7% versus 18.2%). Deep-fried egg pattern was the most common pattern found among suburban participants. Food patterns were associated with residence, dietary intakes except carbohydrate, GI, and GL. The association of food patterns with blood glucose and abnormal blood glucose status was not found in either conventional analysis or survey analysis. Among residents who defined as DM by FBG and 2hPPBG, one- fifth of them had fruit-coffee-sticky pattern.

**Table 5:** Dietary intakes and blood glucose across different types of food patterns

	Milk-sweets-wheat (*n*=53)	Rice-vegetable-meat (*n*=82)	Legume-preserved food (*n*=72)	Snack-soft drink-salted (*n*=77)	Fruit-coffee-sticky (*n*=68)	Deep-fried egg (*n*=88)	*p*	
Residence; *n* (%)							0.004	0.29^a^
Urban	31 (14.1)	50 (22.7)	32 (14.6)	37 (16.8)	40 (18.2)	30 (13.6)		
Suburban	22 (10.0)	32 (14.6)	40 (18.2)	40 (18.2)	28 (12.7)	58 (26.3)		
Dietary intakes								
Calorie (kcal/d), mean (sd)	1319.7 (306.5)	1390.0 (271.1)	1355.1 (241.2)	1388.3 (292.6)	1386.7 (303.2)	1264.1 (259.8)	0.021	
Carbohydrate (g/d), median (IQR)	172.4 (141, 194.4)	173.1 (148.2, 198.4)	167.8 (149.3, 191.7)	164.2 (137.6, 199.3)	180.6 (144.8, 202.3)	163 (143.2, 195.8)	0.808	
Fat (g/d), mean (sd)	42.1 (13.2)	46.5 (15.7)	43.3 (13.4)	48.6 (15.6)	44.6 (15.5)	39.1 (11.1)	<0.001	
Protein (g/d), median (IQR)	42.4 (36.0, 51.4)	46.3 (38.6, 54.0)	50.6 (42.6, 60.2)	47.4 (39.4, 57.5)	48.1 (41.2, 55.6)	45.1 (35.8, 51.3)	0.003	
GI (per day), median (IQR)	60.2 (57.7, 62.2)	61.9 (60.4, 63.2)	60.4 (58.7, 61.9)	58.9 (57.6, 60.9)	60.6 (57.8, 61.8)	62.7 (61.2, 63.6)	<0.001	
GL (per day), median (IQR)	64.6 (56.7, 73.4)	70.7 (62.5, 82.4)	61.7 (52.0, 73.6)	63.6 (54.4, 73.3)	63.0 (53.5, 79.6)	62.2 (49.8, 76.5)	0.002	
Blood glucose								
FBG, median (IQR)	115.4 (104.3, 128.8)	110.4 (102.1, 128.5)	111.0 (103.2, 120.4)	112.1 (103.2, 131.0)	114.3 (106.6, 131.8)	112.1 (105.4, 122.9)	0.782	
2hPP, median (IQR)	126.5 (107.7, 153.2)	144.9 (119.0, 165.1)	133.8 (116.3, 165.9)	136.5 (119.9, 172.1)	137.6 (114.3, 174.8)	143.2 (125.4, 174)	0.227	
Abnormal blood glucose status using fasting blood glucose; *n* (%)							0.775	0.671^a^
DM	7 (9.7)	13 (18.1)	12 (16.7)	13 (18.0)	15 (20.8)	12 (16.7)		
Non-DM	46 (12.5)	69 (18.8)	60 (16.3)	64 (17.4)	53 (14.4)	76 (20.6)		
Abnormal blood glucose status using 2-hour postprandial blood glucose; *n* (%)							0.866	0.748^a^
DM	6 (8.2)	15 (20.6)	13 (17.8)	13 (17.8)	13 (17.8)	13 (17.8)		
Non-DM	47 (12.8)	67 (18.3)	59 (16.1)	64 (17.4)	55 (15.0)	75 (20.4)		

kcal/d=kilocalorie per day; g/d=gram per day; sd=standard deviation; IQR=interquartile range; FBG=fasting blood glucose; 2hPP=2-hour postprandial blood glucose; DM=diabetes mellitus; non-DM=non- diabetes mellitus.

a *p* value obtained from survey analysis.

## Discussion

Dietary intake and food patterns were significantly different between middle-aged participants in urban and suburban areas; even there was no socio-economic difference. Fasting and 2-hour postprandial blood glucose concentrations were highly correlated and were distinguishable from abnormal blood glucose levels. Females predominated in our study samples, similarly to the samples in previous studies in developed and developing nations (10, 22, 31, 32). This finding may be explained by the demographic nature of Myanmar (33) where most housewives are at home, just as in other developing countries (20, 32). When data analysis using a survey package by applying appropriate sampling weights was conducted, the values of mean and standard error obtained were similar to those obtained by conventional analysis.

There is no previous study comparing the dietary intake of middle-aged adults between urban and suburban areas. However, the descriptive information relating dietary intakes in various study settings showed some differences (8, 34–38). The amount of carbohydrate consumed was commonly high among Asians because rice is their main staple food (10, 35, 36, 39). Compared to other studies conducted among Asians and Western populations, the participants in our study consumed lower energy, carbohydrate, and protein but higher fat intake (34–38). Likewise, dietary GI and GL were also lower than those in other Asian studies (7, 10, 17, 40). The low average intake in energy of respondents may be partly due to underreporting of the intake in recording 4-day food diaries. Higher fat, protein, and GL values across various food patterns in urban areas were similar to the food patterns reported in Western countries (8, 36, 38). These findings reflect the impact of urbanization, especially on dietary intake. High GL value in rice-vegetable-meat pattern consumers may be related to the composition of rice in this specific pattern.

A food pattern that combines food intakes and nutrients has become a public health interest (5). A review on 58 articles using food pattern analysis showed that the number of food patterns ranged from 2 to 25, with an explained variance of 15–93%. The food pattern highly loaded with deserts or sweets, similar to our ‘milk-sweets-wheat’ pattern, was the most reproducible one (41). An Asian-like food pattern, the ‘rice-vegetable-meat’ pattern in our study, resembled the ‘Green Water’ dietary pattern of Chinese people (13). Furthermore, the ‘snack-soft drink-salted’ pattern in our study was quite analogous to the ‘more snack and drinks’ pattern of Hong Kong people (7). The remaining three patterns are not comparable to other food patterns as a consequence of the natural variations related with food habits, preferences, and availability in the assorted ethnic and geographic contexts (41).

The previous studies conducted in various populations did not report on food patterns between urban and suburban areas. Differences in food patterns between urban and suburban participants could not be explained by any socio-demographic factors in this study. However, food availability, affordability, and the nutritional knowledge of people were shown to be associated factors in previous studies (42, 43). The association between dietary intake and food pattern was not reported in previous studies. Nevertheless, our study found the diverse amounts in fat, protein, GI, and GL depending on major components of diets across various food patterns (10, 34–39).

To estimate the blood glucose level in the community-based surveys, either venous blood (7, 20, 44) or capillary blood (21), or both capillary and venous blood (1) were applied. Although venous blood glucose levels are recommended by WHO, the fasting capillary blood glucose level can be applied in field research study as an alternative to that of venous blood glucose (28, 29). Our sample mean FBG concentration was higher than the WHO reported value for 2009 (25). The studies on abnormal blood glucose status or diabetes identification conducted in developing countries used various methods including fasting venous glucose (20), fasting capillary glucose (21), Oral Glucose Tolerance Test (OGTT) (32), fasting venous and OGTT combined (7), and self-reported (22, 40). The recommended OGTT requires fasting and 1-hour and 2-hour blood glucose testing after the 75g glucose load (29) in which the sugar solution can cause nausea, vomiting, bloating, or headache in some people (45).

The association between food patterns and abnormal blood glucose status was inconclusive. The studies conducted in Western and Asian countries revealed a high consumption of fruits and vegetables as either a protective factor (11, 12) or a risk factor (13) for impaired glucose metabolism, including DM. In addition, the Western-dietary pattern, characterized by a high intake of meat and processed meat, was positively associated with DM (14, 15). In our study, we did not find any association between food patterns and DM that could support the independent effect of diet on glucose metabolism. The association between food patterns and the abnormal blood glucose status of our participants was reduced, to some extent, after adjusting the sampling design. This may be related with sample size, different methods of dietary assessments, and number of factors studied (11–15, 39). After we had applied the sampling weights, the significant association between food patterns and residence (urban and suburban) disappeared.

To obtain dietary intake and food patterns, a 4-day food diary, which is considered the ‘gold standard’ or ‘reference method’, was applied. Since this approach can minimize a memory or recall bias, it has the potential to provide quantitatively accurate information on consumed food during the recording period (46). There was no study considering dietary intake and blood glucose in urban and suburban people. However, there were some limitations in our study. First, the GI and GL values used in the study were based on the values estimation from the International table of GI and GL values: 2002. Second, our study samples were not stratified by gender, although dietary intake and food patterns may differ between males and females (47). Moreover, no association between intakes (expressed as food groups, macronutrients, and GI or GL) and blood glucose levels may be explained by random errors in dietary recall, which would be rarely inevitable in nutritional epidemiologic studies (48).

## Conclusion

In conclusion, there was no difference in the blood glucose between middle-aged adults in urban and suburban areas, though high-risk dietary intakes were found in urban areas. Highly correlated 2-hour postprandial and FBG had the ability to distinguish abnormal blood glucose status. This finding may suggest the application of 2-hour postprandial blood glucose for DM screening in resource-limited low-income countries where fasting or OGTT is not feasible. A population-based study with a large sample is required to identify the association between abnormal blood glucose status and dietary factors.
